# Unicompartmental Knee Arthroplasty for Knee Osteoarthritis With the Pellegrini–Stieda Lesion: A Case Report

**DOI:** 10.3389/fsurg.2022.922896

**Published:** 2022-07-06

**Authors:** Qiuyuan Wang, Wanshou Guo, Zhencai Shi, Weiguo Wang, Qidong Zhang

**Affiliations:** ^1^ Graduate School of Beijing University of Chinese Medicine, Beijing, China; ^2^ Department of Orthopaedic Surgery, China–Japan Friendship Hospital, Beijing, China

**Keywords:** unicompartmental knee arthroplasty, Pellegrini–Stieda lesion, knee osteoarthritis, ossification, medial collateral ligament (MCL)

## Abstract

Pellegrini–Stieda lesion is described as ossification on the origin of medial collateral ligament. We present a case of end-stage knee osteoarthritis with the Pellegrini–Stieda lesion treated by unicompartmental knee arthroplasty (UKA). During the postoperative follow-up, an interesting change occurred such that the ossification lesion disappeared gradually and did not relapse. It is supposed that the disappearance was caused by UKA changing the abnormal biomechanics of the varus osteoarthritic knee.

## Introduction

Pellegrini–Stieda lesion is usually described as ossification on the origin of medial collateral ligament (MCL) (1–5). Its clinical significance was first studied and reported by Pellegrini in 1905 and then by Stieda in 1908 (6, 7). The mechanism of the Pellegrini–Stieda lesion was found to be associated with trauma (4, 8–10). Conservative therapies are usually adopted for curing the lesion (11–13), and surgical cases with good outcomes have also been reported in the literature (14). This article reports the case of end-stage knee osteoarthritis with the Pellegrini–Stieda lesion treated by unicompartmental knee arthroplasty.

## Case Presentation

**Figure 1:**
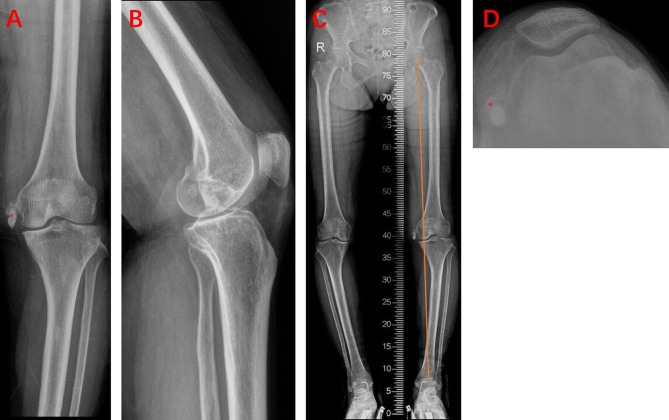
Preoperative x-ray of the left knee. (**A**) AP view; (**B**) lateral view; (**C**) full-weight bearing view; (**D**) merchant view. *Pellegrini–Stieda lesion.

**Figure 2:**
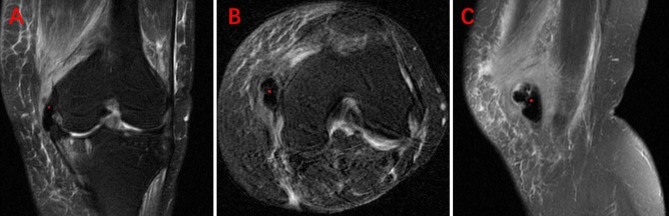
Preoperative MRI of the left knee. (**A**) Coronal T2 weighted image (T2WI); (**B**) transverse axis proton density weighted image (PDWI); (**C**) sagittal T2WI. *Pellegrini–Stieda lesion.

A 76-year-old woman was hospitalized for acute exacerbation pain and activity limitation of the left knee. Over 10 years ago, she started to feel pain in her left knee. The pain often worsened after long-time weight-bearing activity and could be relieved by rest. She always accepted conservative treatment (oral and external-used non-steroidal anti-inflammatory drugs, physical therapy, and acupuncture) and received a fair curative effect. Five days back, her left knee pain unexpectedly worsened, accompanied by joint swelling and a sense of knee joint locking. She experienced difficulty with climbing stairs and her pain-free walking distance was less than 100 m. A physical examination of the left knee showed tenderness on the medial side, especially on the medial joint space, a weak positive floating patella test, and a 0–5° to 100° range of motion (ROM). The HSS score of the left knee was 55. An x-ray of the left knee indicated a degenerative lesion in the medial knee compartment, presented as narrowed joint space and subchondral sclerosis (Figure 1A). A strip-shaped high-density shadow in the medial is noticeable (Figures 1A, C). Further MRI examination of the left knee showed ossification involving medial collateral ligament (MCL) and medial joint capsule (Figure 2). The ossification was considered as the Pellegrini–Stieda lesion.

**Figure 3:**
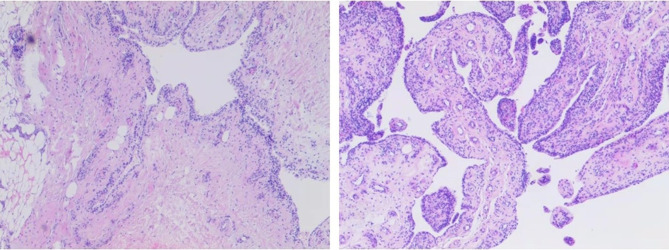
Pathology of the synovium.

**Figure 4:**
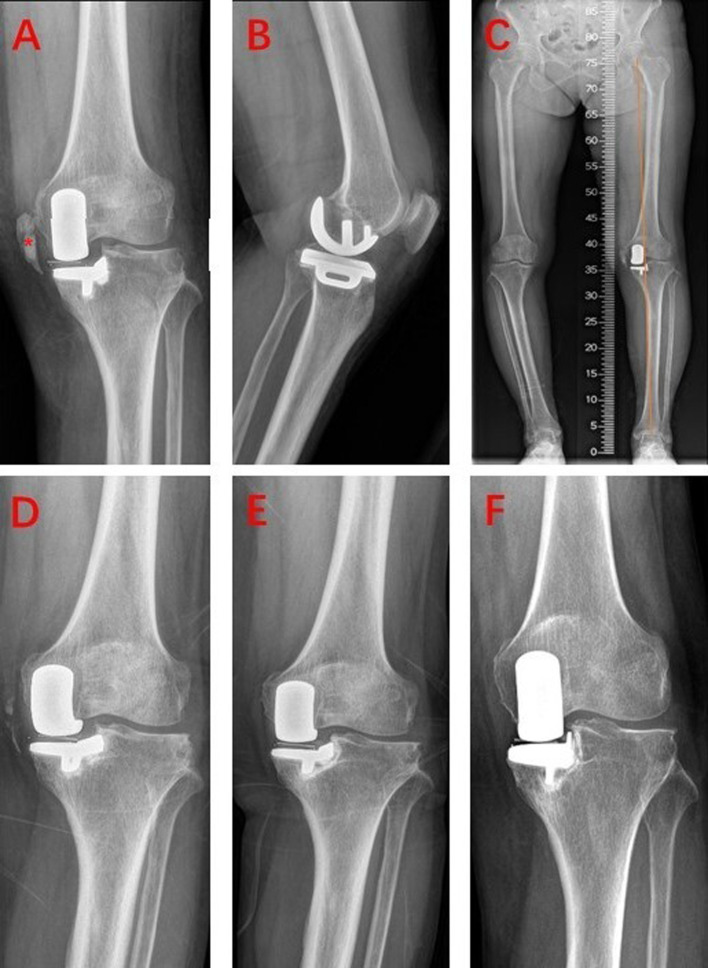
Postoperative x-ray during discharge and follow-up. (**A**–**C**) X-ray at discharge, *Pellegrini–Stieda lesion; (**D**) postoperative 3 months; (**E**) postoperative 9 months; (**F**) postoperative 3.5 years.

According to the anteroposterior and lateral view of the x-ray, the cartilage wear area was mainly located in the anteromedial of the left knee (Figures 1A, B). The full-weight-bearing x-ray showed varus deformity of the left knee, and the intra-articular deformity occupied the main (Figure 1D). So, we conducted medial mobile-bearing unicompartmental knee arthroplasty (Oxford UKA, Zimmer Biomet, Warsaw, IN, United States) for this patient. Intraoperatively, we found cartilage injury on the medial tibial plateau and medial femoral condyle (Outerbridge grade III). The synovium around the medial femoral condyle presented hyperemia and edema. The pathology of the synovium found villous hyperplasia and indicated acute and chronic inflammation (Figure 3). The surgery progressed well, and the patient returned to the ward safely after recovery from anesthesia. Then, she was treated with common therapies such as analgesia, ice compress, infection prevention, early functional exercises, etc. Her postoperative recovery was good, and the ROM was 0-0–110° on discharge. The postoperative x-ray indicated that the varus deformity was corrected, and the position of the prosthesis was satisfactory **(**Figures 4A–C).

However, the x-ray during follow-up showed a surprising finding. The postoperative x-ray during hospitalization showed that the medial ossification lesion still existed and was almost the same as that of preoperative x-ray. However, the postoperative 3-month x-ray showed that the ossification lesion shrank obviously and became discontinuous (Figure 4D). Nine months after surgery, the x-ray found that the ossification lesion entirely disappeared (Figure 4E). The latest outpatient follow-up was on postoperative 3.5 years, and no relapse of the ossification lesion was found on the x-ray (Figure 4F). The patient was satisfied with her left knee function, and the HSS score was 90 at the last follow-up.

## Discussion

To our knowledge, this is the first case report of UKA treating end-stage osteoarthritis with the Pellegrini–Stieda lesion. The most valuable finding was that the ossification lesion disappeared gradually after UKA and did not relapse during the follow-up. We suppose this interesting change may be related to the improvement in the biomechanical environment induced by UKA. As UKA can restore the tension of MCL and correct the varus deformity (15–17), the abnormal biomechanics of the osteoarthritic knee was reversed (18, 19). Of course, the mechanism behind this change is highly complex, and more studies are required on this special and interesting clinical finding.

The anatomic location of the Pellegrini–Stieda lesion has always been controversial. Pellegrini described that the lesion was located at the origin of knee MCL according to the findings of surgical removal (6). While, according to the cadaver study, Stieda held a different opinion that the lesion was located medial to the origin of the gastrocnemius medial head muscle (7). In subsequent studies and reports, the Pellegrini–Stieda lesion can also involve the adductor magnus tendon and posterior attachment of medial patellofemoral ligament (MPFL) (5, 14, 20). From the MRI of our case, the ossification lesion involved MCL and medial joint capsule. So, the characteristics of this case fit the descriptions of the Pellegrini–Stieda lesion in the literature. Mendes et al. proposed a classification for the Pellegrini–Stieda lesion on the basis of anatomic and clinical studies (5). According to their description, our case belongs to type I for representing a beak-like appearance with an inferior orientation and femoral attachment.

With regard to surgical techniques, we followed the basic principles of Oxford UKA that the balance of flexion and extension gap was achieved only by osteotomy and the tension of MCL was recovered by the filling the mobile bearing into the joint space. Any procedure on MCL may affect the soft tissue balance. Therefore, no release on MCL or any intervention on the ossification lesion was performed.

The characteristic of medical history is another noteworthy point of this case. The patient complained that her left knee pain had been presented for over 10 years and the pain became acute 5 days before hospitalization. Although her pain could perhaps be attributed to osteoarthritis, the real reason for its worsening is worth analyzing, as the symptomatic progression of osteoarthritis is usually chronic (21). Although uncommon, an acute inflammatory state of osteoarthritis is highly possible for this case. But our speculation suggests that the acute pain was mainly due to the impingement between the ossification lesion and synovium, which could lead to synovial interposing and acute synovitis. The pathologic results also testify to this. Due to the varus knee deformity, the load of medial knee compartment became heavier, and the tension of MCL also decreased (22). In this condition, it is possible to generate impingement between the ossification lesion and the surrounding synovium. While, after UKA, the hyperplastic arthritic synovium was removed, the worn medial compartment was replaced, and the tension of MCL was also recovered. As a result, the patient's pain was released.

## Conclusion

This article presents a case of end-stage knee osteoarthritis with the Pellegrini–Stieda lesion treated by UKA. Surprisingly, the ossification lesion disappeared gradually during the post-operative follow-up and did not relapse. It was supposed that the disappeared ossification was due to UKA reserving the abnormal biomechanics of the varus osteoarthritic knee. More studies are required to investigate this phenomenon.

## Data Availability

The raw data supporting the conclusions of this article will be made available by the authors, without undue reservation.
